# Interactions between inflammatory signals and the progesterone receptor in regulating gene expression in pregnant human uterine myocytes

**DOI:** 10.1111/j.1582-4934.2012.01567.x

**Published:** 2012-09-26

**Authors:** Yun Lee, Suren R Sooranna, Vasso Terzidou, Mark Christian, Jan Brosens, Kaisa Huhtinen, Matti Poutanen, Geraint Barton, Mark R Johnson, Phillip R Bennett

**Affiliations:** a Parturition Research Group Imperial College London, Institute of Reproductive and Developmental BiologyLondon, UK; b Department of Physiology Institute of Biomedicine, University of TurkuTurku, Finland; c Turku Center for Disease Modeling, University of TurkuTurku, Finland; d Department of Bioinformatics, Imperial College LondonLondon, UK

**Keywords:** labour, parturition, progesterone, PR, inflammation, NF-kappaB

## Abstract

The absence of a fall in circulating progesterone levels has led to the concept that human labour is associated with ‘functional progesterone withdrawal’ caused through changes in the expression or function of progesterone receptor (PR). At the time of labour, the human uterus is heavily infiltrated with inflammatory cells, which release cytokines to create a ‘myometrial inflammation’ *via* NF-κB activation. The negative interaction between NF-κB and PR, may represent a mechanism to account for ‘functional progesterone withdrawal’ at term. Conversely, PR may act to inhibit NF-κB function and so play a role in inhibition of myometrial inflammation during pregnancy. To model this inter-relationship, we have used small interfering (si) RNA-mediated knock-down of PR in human pregnant myocytes and whole genome microarray analysis to identify genes regulated through PR. We then activated myometrial inflammation using IL-1β stimulation to determine the role of PR in myometrial inflammation regulation. Through PR-knock-down, we found that PR regulates gene networks involved in myometrial quiescence and extracellular matrix integrity. Activation of myometrial inflammation was found to antagonize PR-induced gene expression, of genes normally upregulated *via* PR. We found that PR does not play a role in repression of pro-inflammatory gene networks induced by IL-1β and that only MMP10 was significantly regulated in opposite directions by IL-1β and PR. We conclude that progesterone acting through PR does not generally inhibit myometrial inflammation. Activation of myometrial inflammation does cause ‘functional progesterone withdrawal’ but only in the context of genes normally upregulated *via* PR.

## Introduction

Progesterone is considered to play a major role in the maintenance of pregnancy. In 1956 Csapo proposed that the essential role of progesterone in pregnancy is to ‘block’ myometrial contractility and that the onset of labour therefore requires withdrawal of this progesterone block. In the majority of mammals labour is preceded by a decline in circulating progesterone concentrations, however, in the human, there is no dramatic fall in progesterone concentrations prior to labour, yet parturition can be induced using progesterone antagonists. This has led to the concept that, in the human, labour is associated with ‘functional progesterone withdrawal’ caused through changes in the expression or function of PR rather than through changes in circulating concentrations of progesterone [1, 2].

The genomic effects of progesterone upon target tissues are generally mediated through nuclear PR. The major PR isoforms, PR-A and PR-B, are encoded from a single gene by differential promoter usage. PR-A, a 94 kD protein, lacks the first N-terminal 164 amino acids of PR-B, which is a 116 kD protein with an additional activation function (AF-3) [1, 2]. In myometrial cells, PR-A has limited transactivation properties at classical progesterone response elements and has been shown to transrepress PR-B and other class I nuclear receptor family members [3, 4]. The PR gene also contains additional putative downstream translational start sites predicted to encode proteins of approximately 60–70 and 39 kD respectively. A 60 kD PR variant found in T47D cells has been termed PR-C. This contains a ligand-binding domain, but lacks a DNA binding domain and has been reported to be cytosolic rather than nuclear [5].

A current theory for the mechanism of ‘functional progesterone withdrawal’ in human myometrium involves a change in the abundance of PR isoforms, leading to a decrease in the ratio of PR-B to PR-A and/or PR-C [1, 2, 4]. Other proposed mechanisms include a decline in the myometrial levels of PR coactivators at term [6], and a decrease in the circulating concentration of bioactive progesterone metabolites associated with decreased steroid 5 beta-reductase expression in the uterus [7].

A further potential mechanism for ‘functional progesterone withdrawal’ in the human involves the transcriptional antagonism between the activated PR and inflammatory signal intermediates, especially the inflammation-associated transcription factor NF-kappaB (NF-κB). A central role for NF-κB in murine parturition has been demonstrated [8]. Nuclear translocation of the p50 and p65 subunits of NF-κB increases in the pregnant mouse uterus towards term and intra-amniotic injection of the NF-κB inhibitor peptide SN50 delays in the onset of labour. Surfactant protein-A (SP-A), secreted by the maturing foetus lung in increasing amounts towards term, was shown to enhance p65 nuclear levels and to induce labour. It has been proposed that SP-A triggers the onset of labour at term by inducing the migration of macrophages to the maternal uterus, where local inflammatory signals activate NF-κB signalling in myocytes, resulting in the stimulation of uterine contractility. Direct protein-protein interaction between p65 and PR, resulting in reciprocal functional antagonism, has been demonstrated in breast cancer cells [9], and may also operate in uterine cells [10, 11].

Activation of myometrial inflammation, or more specificially of the NF-κB pathway in the myometrium appears to be an attractive mechanism to account for ‘functional progesterone withdrawal’ at term. However, the functional consequences of the interaction between PR and NF-κB on myometrial gene expression in pregnancy has not yet been determined. We have therefore combined small interfering (si)RNA-mediated knock-down of PR in human term pregnant myocytes with whole genome microarray analysis to identify those genes regulated by activated PR. We then used IL-1β stimulation, which activates NF-κB and induces myometrial inflammation, to determine the role of PR in the transcriptional regulation of myometrial inflammation.

## Materials and methods

### Tissue collection and cell culture

**Fig 1:**
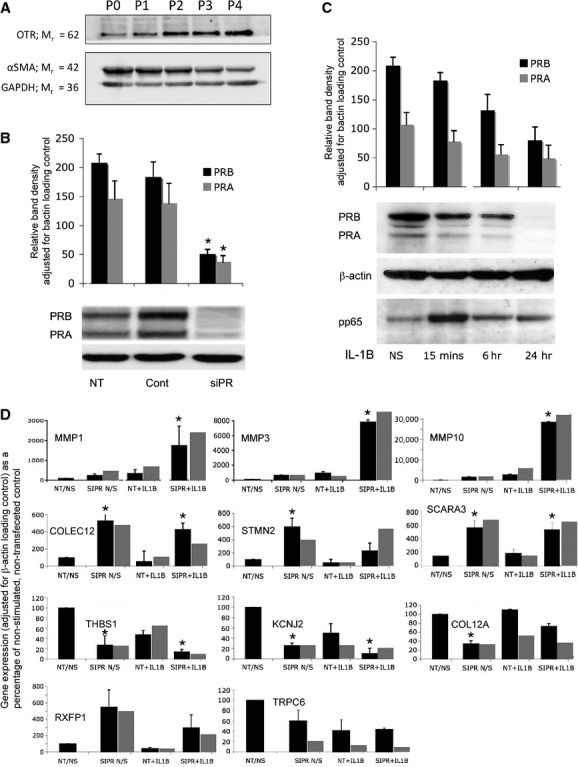
(A) Expression of oxytocin receptor (OTR), alpha-smooth muscle actin (aSMA) and glyceraldehyde 3-phosphate dehydrogenase (GAPDH) at passage numbers zero through four. (B) Expression of PRB and PRA measured by Western analysis in cultured human myocytes in non-transfected (control), non-targeting siRNA transfected (NT) and PR targeting siRNA transfected (siPR) cells. Blots were scanned for densitometric analysis, values were normalized for GAPDH and are expressed as arbitrary units. One example blot is shown. Graphs *n* = 6 ± S.E., *P < 0.05 compared with control. (C) Expression of PRB and PRA and of Ser536-P-p65 (NF-kappaB p65) measured by Western analysis in cultured human myocytes in controls incubated with MPA (100 nM) (control) and following incubation with MPA (100 nM) and IL1B (1 ng/ml) for up to 24 hrs. Blots were scanned for densitometric analysis, values were normalized for GAPDH and are expressed as arbitrary units. One example blot is shown. Graphs *n* = 4 ± S.E. *P < 0.05 compared with control. (D) Validation experiments measuring expression of selected genes in myocytes in culture following siRNA knock-down of PR (siPR N/S), incubation with and without IL1B (1 ng/ml) (NT + IL1B), or both together (siPR + IL1B), compared with non-targeting siRNA transfected (NT) control. Black bars show qRT-PCR validation data (*n* = 4 ± S.E. *P < 0.05 compared with control). Grey bars show data from microarray for comparison.

Tissue was collected with local ethics committee approval and informed consent from patients at Queen Charlotte's and Chelsea Hospital, London. Myometrial tissue was obtained from the upper edge of lower uterine incision, made at the location of the bladder fold at the time of Caesarean section. Elective caesarean section was performed in each case at or after 39 weeks in uncomplicated pregnancies. Indications were breech presentation and previous caesarean section. Myometrial tissue was minced and digested for 45 min. in DMEM with 1 mg/ml collagenase type IA and IX (Sigma-Aldrich Company Ltd, Poole, UK). Cells were centrifuged at 400 × *g* for 10 min. and grown in DMEM with 10% foetal calf serum, l-glutamine and penicillin-streptomycin (37°C and 5% CO_2_)_._ Cells (passage number 3 or 4) were incubated with 100nM medroxyprogesterone acetate (MPA) in 2% reduced serum for 24 hrs before IL-1β (R&D Systems, Europe Ltd., Abingdon, UK) was added to a final concentration of 1 ng/ml for 6 hrs. To confirm that the cells established in our cultures are myocytes and not fibroblasts or epithelial cells, we undertook Western analysis for alpha-smooth-muscle actin, and oxytocin receptor and found no significant changes in expression between passages 0–4 (Fig. 1A).

### Transfection of siRNA

ON-TARGETplus SMART pool human RNA (Dharmacon, Lafayette, CO, USA) was used to knock-down PR. SiGLO (Dharmacon) was used as a positive control, giving a high transfection efficiency of approximately 90%, and ON-TARGETplus Non-Targeting Pool (Dharmacon) was used as a negative control. The siRNAs were transfected using Dharmafect 2 (Dharmacon) transfection reagent at a final concentration of 20 μM according to manufacturer's instructions.

### Protein extraction

Myocytes were scraped into buffer A [10 mM HEPES pH 7.4, 10 mM KCl, 0.1 mM EDTA, 0.1 mM EGTA, 2 mM DTT, complete protease inhibitor tablets (Roche, Welwyn Garden City, UK) and 2 M KOH to adjust pH to 7.4]. The resulting suspension was collected and incubated on ice for 20 min. To the incubated mixture, NP-40 (Nonidet P-40, Sigma-Aldrich Company Ltd) was added to give a final concentration of 1% and carefully mixed. The cytosolic protein fraction was extracted by centrifuging at 13,000 × *g* for 30 sec. The pellet was then resuspended in buffer B (10 mM HEPES, 10 mM KCl, 0.1 mM EDTA, 0.1 mM EGTA, 2 mM DTT, 400 mM NaCl, 1% NP-40) and complete protease inhibitor tablets (Roche) to lyse the nuclear membrane. Samples were incubated on a shaking platform for 15 min. Cell suspensions were centrifuged at 13,000 × *g* for 5 min. and supernatant (nuclear protein fraction) was collected, snap-frozen and stored at −80°C for later use.

### Western blot analysis

A quantity of 50μg protein samples were mixed with Laemmli sample buffer (1:1) containing beta-mercaptoethanol (5%), and boiled for 5 min. Protein was separated by SDS-PAGE gels and transferred onto nitrocellulose membrane (Amersham Biosciences, Amersham, UK). The membrane was blocked in buffer containing 5% milk powder, PBS and 0.1% Tween 20 for 30 min., and immunoblotted with primary antibody for 1 hr in 1% milk buffer followed by secondary antibody for 45 min. Horseradish peroxidase-conjugated secondary antibody (Santa Cruz Biotechnology, Heidelberg, Germany) was used with ECL Plus (Amersham Biosciences) chemiluminescent reagents for signal detection. Antibodies used were PR Novocastra NCL-L-PGR-312 (Novocastra Laboratories Ltd, Newcastle upon Tyne, UK) OTR Santa Cruz SC-8102, alpha smooth muscle actin Sigma A2547, GAPDH Millipore MAB374, Millipore, Watford, UK, beta actin Abcam ab8226 (Abcam, Cambridge, UK) and NFkB pp65 Serine 536 Cell Signalling 3033S (Cell Signaling Technology, New England Biolabs, Hitchin, UK).

### RNA extraction

Total RNAs were isolated using Trizol (Invitrogen, Paisley, UK) according to the manufacturer's protocol and further purified using the RNeasy mini Kit (QIAGEN, Crawley, UK). RNA integrity and purity were assessed using a Bioanalyzer 2100 (Agilent Technologies Inc., Santa Clara, CA, USA).

### Quantitative real time polymerase chain reaction (RTQ-PCR)

Total RNA 1 μg was reverse transcribed with oligo dT random primers using MuLV reverse transcriptase (Applied Biosystems Ltd, Carlsbad, CA, USA). Paired oligonucleotide primers for amplification of genes of interest were designed using Primer 3 software (http://www.ncbi.nlm.nih.gov/tools/primer-blast/) against the sequences downloaded from GenBank. The primer sets used (Table 1) produced amplicons of the expected size and where possible flanked intron/exon junctions. Quantitative PCR was performed using Power SYBR Green PCR master mix (Applied Biosystems Ltd.,) and amplicon yield was monitored during cycling in a RotorGene Sequence Detector (Corbett Research Ltd.) that continually measures fluorescence caused by the binding of the dye to double-stranded DNA. Pre-PCR cycle was 10 min. at 95°C followed by up to 45 cycles of 95°C for 20 sec., 58–60°C for 20 sec. and 72°Cfor 20 sec. followed by an extension at 72°C for 15 sec. The final procedure involves a melt over the temperature range of 72–99°C rising by 1 degree steps with a wait for 15 sec. on the first step followed by a wait of 5 sec. for each subsequent step. The cycle at which the fluorescence reached a preset threshold (cycle threshold) was used for quantitative analyses. The cycle threshold in each assay was set at a level where the exponential increase in amplicon abundance was approximately parallel between all samples. mRNA data were expressed relative to the amount of the constitutively expressed housekeeping gene, GAPDH.

**Table 1:** Sequences for PCR primers used in quantitative RT-PCR validation studies

Gene symbol	Forward primer (5′–3′)	Reverse primer (5′–3′)
MMP1	CAGAGGGAGCTTCCTAGCTG	AGCTGTGCATACTGGCCTTT
MMP3	CACTCACAGACCTGACTCGG	AGTCAGGGGGAGGTCCATAG
MMP10	CAAAATCTGTTCCTTCGGGA	TCTCCCCTCAGAGTGCTGAT
COLEC12	CAGCCAGCTCAACTCATTCA	GGTCTTTCAGGTTCTGCTCG
STMN2	AAGAAAGTCTCAGGAGGCCC	TGTTGTTCTCCTCCAAAGCC
SCARA3	AGGAGAGGGCAGAGGAAGAC	TGCCCAGAGACAGATGTGAG
THBS1F	ACCAAAGCCTGCAAGAAAGA	TCTGTACCCCTCCTCCACAG
KCNJ2	CGCTTTTTACAAACCACTGGA	AACATGTCCTGTTGCTGGC
COL12A1	TGGAAAATCCCAGGATGAAG	CAGCTTTAATGCCCAAGGAG
RXFP1	TCACCTCAGTCGAATTTCCC	TCAGGTAAACGGGTGAGGAC
TRPC6	AGAAGTCGAGGCCATTCTGA	GAACTTGACCGCCATTGTCT
PR	AGCCCACAATACAGCTTCGAG	TTTCGACCTCCAAGGACCAT
GAPDH	TGATGACATCAAGAAGGTGGTGAAG	TCCTTGGAGGCCATGTAGGCCAT

### Affymetrix HgU133 Plus 2.0 array processing

RNA Samples were profiled by the Finnish DNA-Microarray Centre using the Affymetrix HgU133 Plus 2.0 GeneChip® (Affymetrix Inc., Santa Clara, CA, USA). Briefly, cDNA was generated from 2 μg of total RNA using the GeneChip® Expression 3′-Amplification One-Cycle cDNA Synthesis kit, in conjunction with the GeneChip® Eukaryotic PolyA RNA Control Kit (Affymetrix Inc.). The cDNA was cleaned up using the GeneChip® Sample Cleanup Module and subsequently processed to generate biotin-labelled cRNA using the GeneChip® Expression 3′-Amplification IVT Labelling Kit (Affymetrix Inc.). A quantity of 25 μg of labelled cRNA was fragmented using 5X fragmentation buffer and Rnase-free water at 94°C for 35 min. A quqntity of 15 μg of the fragmented, biotin-labelled cRNA was made up in a hybridization cocktail and hybridized to the HgU133 Plus 2.0 array at 45°C for 16 h. Following hybridization the arrays were washed and stained using the Affymetrix Fluidics Station 450 and scanned using the Affymetrix GeneChip® Scanner 3000. All steps of the process were quality controlled by measuring yield (μg), concentration (μg/l) and 260:280 ratios *via* spectrophotometry using the Nanodrop ND-1000 and sample integrity using the Agilent 2100 bioanalyser (Agilent Technologies Inc.).

## Results

### PR regulates gene networks involved in myometrial quiescence and extracellular matrix integrity

To identify genes regulated in human myometrium by liganded PR, primary myocyte cultures were transfected with siRNA targeting PR or non-targeting control siRNA, and then incubated with medroxyprogesterone acetate (MPA; 100 nM) for 24 hrs. Cells were lysed, mRNA extracted and subjected to genome-wide expression profiling using Affymetrix HgU133 Plus 2.0 GeneChip® (Affymetrix Inc.). Targeting PR using siRNA resulted in greater than 80% knock-down of PR-B and PR-A expression at protein level on Western analysis (Fig. 1B).

The microarray data were filtered for significant changes in expression (*P* < 0.05 after correction for multiple testing). Overall, 354 probe sets showed a significant change in expression upon PR-knock-down. Of these, 150 probes sets were increased and 204 decreased. When unannotated sequences and duplicate probe sets were eliminated, 159 (57%) and 118 (43%) unique gene sequences were increased and decreased, respectively, upon knock-down of PR. A total of 44 genes were ≥2-fold downregulated, of which only 11 transcripts were inhibited by ≥3-fold (Table 2). As PR-knock-down decreased the expression of these genes, they represent genes upregulated in a PR-dependent manner.

**Table 2:** Unique genes whose expression was decreased by PR-knock-down, and whose expression is therefore upregulated by PR in the presence of progesterone. 44 genes showed changes in expression of 2-fold or more. 11 genes showed changes in expression of 3-fold or more. In each case *n* = 4, *P* < 0.0005, Adjusted *P* < 0.05)

ID	Gene symbol	Gene title	Fold change in expression
222853_at	FLRT3	Fibronectin leucine-rich transmembrane protein 3	−5.419
217287_s_at	TRPC6	Transient receptor potential cation channel C6	−5.406
202708_s_at	HIST2H2BE	Histone cluster 2, H2be	−4.98
223877_at	C1QTNF7	C1q and tumour necrosis factor related protein 7	−4.545
1559114_a_at	CXCR7	CXC chemokine receptor type 7	−4.348
206765_at	KCNJ2	Potassium inwardly rectifying channel, subfamily J, member 2	−4.138
239710_at	FIGN	Fidgetin	−4.069
201107_s_at	THBS1	Thrombospondin 1	−3.921
235371_at	GLT8D4	Glycosyltransferase 8 domain containing 4	−3.448
231186_at	FLJ43390	Hypothetical LOC646113	−3.196
202016_at	MEST	Mesoderm specific transcript homologue	−3.153
233109_at	COL12A1	Collagen type XII alpha-1 precursor	−2.869
226610_at	CENPV	Centromere protein V	−2.781
204837_at	MTMR9	Myotubularin related protein 9	−2.748
225008_at	ASPH	Aspartyl(asparaginyl)beta-hydroxylase	−2.612
201487_at	CTSC	Cathepsin C	−2.411
238447_at	RBMS3	RNA binding motif, single stranded interacting protein	−2.41
222771_s_at	MYEF2	Myelin expression factor 2	−2.402
211959_at	IGFBP5	Insulin-like growth factor binding protein 5	−2.384
220153_at	ENTPD7	Ectonucleoside triphosphate diphosphohydrolase 7	−2.383
224613_s_at	DNAJC5	DnaJ (Hsp40) homologue, subfamily C, member 5	−2.369
208903_at	RPS28	Ribosomal protein S28	−2.367
224767_at	RPL37	Ribosomal protein L37, mRNA	−2.357
205807_s_at	TUFT1	Tuftelin 1	−2.342
224984_at	NFAT5	Nuclear factor of activated T-cells 5, tonicity-responsive	−2.335
222582_at	PRKAG2	Protein kinase, AMP-activated, gamma 2 non-catalytic subunit	−2.314
223155_at	HDHD2	Haloacid dehalogenase-like hydrolase domain containing 2	−2.311
202091_at	ARL2BP	ADP-ribosylation factor-like 2 binding protein	−2.284
213223_at	RPL28	Ribosomal protein L28	−2.236
213024_at	TMF1	TATA element modulatory factor 1	−2.217
205012_s_at	HAGH	Hydroxyacylglutathione hydrolase	−2.196
1554489_a_at	CEP70	Centrosomal protein 70 kD	−2.17
218888_s_at	NETO2	Neuropilin (NRP) and tolloid (TLL)-like 2	−2.163
229456_s_at	DDAH1	Dimethylarginine dimethylaminohydrolase 1 (DDAH1) V2	−2.154
203854_at	CFI	Complement factor I	−2.128
204042_at	WASF3	WAS protein family, member 3	−2.122
215506_s_at	DIRAS3	DIRAS family, GTP-binding RAS-like 3	−2.116
201285_at	MKRN1	Makorin ring finger protein 1	−2.093
212875_s_at	C2CD2	C2 calcium-dependent domain containing 2	−2.084
224727_at	C19orf63	Chromosome 19 open reading frame 63	−2.082
209066_x_at	UQCRB	Ubiquinol-cytochrome c reductase binding protein	−2.027
224559_at	MALAT1	Metastasis associated lung adenocarcinoma transcript 1	−2.01
205381_at	LRRC17	leucine rich repeat containing 17	−2.008
205755_at	ITIH3	Inter-alpha (globulin) inhibitor H3	−2.003

Of the 159 genes upregulated in response to PR knock-down, 79 increased by ≥2-fold, including 24 that were induced ≥3-fold (Table 3). As PR silencing upregulated their expression, these genes represent transcriptional targets actively repressed by PR.

**Table 3:** Unique genes whose expression was increased by PR-knock-down, and whose expression is therefore inhibited by liganded-PR. 24 genes showed changes in expression of 3-fold or more. 55 genes showed changes in expression of 2 to 3-fold. (In each case *n* = 4, *P* < 0.0005, Adjusted *P* < 0.05)

ID	Gene symbol	Gene title	Fold change in expression
205680_at	MMP10	Matrix metallopeptidase 10 (stromelysin 2)	7.617
215867_x_at	CA12	Carbonic anhydrase XII	7.571
223843_at	SCARA3	Scavenger receptor class A, member 3	6.989
214799_at	NFASC	Neurofascin homologue (chicken)	6.311
228155_at	C10orf57 58	Chromosome 10 open reading frame 57 and 58	5.803
203001_s_at	STMN2	Stathmin-like 2	5.511
244353_s_at	SLC2A12	Solute carrier family 2 (facilitated glucose transporter), member 12	5.277
221019_s_at	COLEC12	Collectin sub-family member 12	5.205
214844_s_at	DOK5	Docking protein 5	4.276
204638_at	ACP5	Acid phosphatase 5, tartrate resistant	4.16
207016_s_at	ALDH1A2	Aldehyde dehydrogenase 1 family, member A2	3.915
217525_at	OLFML1	Olfactomedin-like 1	3.669
220351_at	CCRL1	Chemokine (C-C motif) receptor-like 1	3.633
213415_at	CLIC2	Chloride intracellular channel 2	2.95
202580_x_at	FOXM1	Forkhead box M1	2.929
209159_s_at	NDRG4	NDRG family member 4	2.862
213001_at	ANGPTL2	Angiopoietin-like 2	2.841
243956_at	SUSD3	MRNA; cDNA DKFZp434J1812	2.787
235198_at	OSTM1	Osteopetrosis associated transmembrane protein 1	2.768
205347_s_at	MGC39900	Thymosin beta15b///thymosin beta 15a	2.705
220911_s_at	KIAA1305	KIAA1305	2.613
228890_at	ATOH8	Atonal homologue 8 (Drosophila)	2.585
228436_at	KCNC4	Potassium voltage-gated channel, Shaw 4	2.578
219937_at	TRHDE	Thyrotropin-releasing hormone degrading enzyme	2.578
1558537_x_at	ZNF844	Zinc finger protein 844	2.564
1569157_s_at	ZNF846	Zinc finger protein 846	2.564
218211_s_at	MLPH	Melanophilin	2.555
226145_s_at	FRAS1	Fraser syndrome 1	2.513
209539_at	ARHGEF6	Rac/Cdc42 guanine nucleotide exchange factor 6	2.504
213836_s_at	WIPI1	WD repeat domain, phosphoinositide interacting 1	2.462
205730_s_at	ABLIM3	Actin binding LIM protein family, member 3	2.446
209197_at	SYT11	Synaptotagmin XI	2.444
1552651_a_at	RFFL	Ring finger and FYVE-like domain containing 1	2.423
235746_s_at	PLA2R1	Phospholipase A2 receptor 1, 180 kD	2.414
241723_at	IQGAP2	IQ motif containing GTPase activating protein 2	2.412
216603_at	SLC7A8	Solute carrier family 7 (cationic amino acid transporter, y+ system), 8	2.407
210768_x_at	TMCO1	Transmembrane and coiled-coil domains 1	2.398
227048_at	LAMA1	Laminin, alpha 1	2.394
204236_at	FLI1	Friend leukaemia virus integration 1	2.393
1563753_at	LOC149684	Hypothetical protein LOC149684	2.372
205113_at	NEFM	Neurofilament, medium polypeptide	2.37
204675_at	SRD5A1	Steroid-5-alpha-reductase, alpha polypeptide 1	2.368
209365_s_at	ECM1	Extracellular matrix protein 1	2.364
211343_s_at	COL13A1	Collagen, type XIII, alpha 1	2.351
204797_s_at	EML1	Echinoderm microtubule associated protein like 1	2.34
224800_at	WDFY1	WD repeat and FYVE domain containing 1	2.327
228438_at	LOC100132891	Homo sapiens hypothetical protein LOC100132891	2.309
203435_s_at	MME	Membrane metallo-endopeptidase	2.302
210291_s_at	ZNF174	Zinc finger protein 174	2.29
232636_at	SLITRK4	SLIT and NTRK-like family, member 4	2.252
207463_x_at	PRSS3	Protease, serine, 3	2.251
213562_s_at	SQLE	Squalene epoxidase	2.248
228728_at	C7orf58	Chromosome 7 open reading frame 58	2.241
225412_at	TMEM87B	Transmembrane protein 87B	2.227
226106_at	RNF141	Ring finger protein 141	2.201
208703_s_at	APLP2	Amyloid beta (A4) precursor-like protein 2	2.197
223395_at	ABI3BP	ABI family, member 3 (NESH) binding protein	2.191
244881_at	LMLN	Leishmanolysin-like (metallopeptidase M8 family)	2.18
213309_at	PLCL2	Phospholipase C-like 2	2.18
205603_s_at	DIAPH2	Diaphanous homologue 2 (Drosophila)	2.164
203666_at	CXCL12	Chemokine (C-X-C motif) ligand 12	2.137
227188_at	C21orf63	Chromosome 21 open reading frame 63	2.084
228253_at	LOXL3	Lysyl oxidase-like 3	2.083
213725_x_at	XYLT1	Xylosyltransferase I	2.053
230256_at	C1orf104	Chromosome 1 open reading frame 104, mRNA	2.047
1558680_s_at	PDE1A	Phosphodiesterase 1A, calmodulin-dependent	2.021
205205_at	RELB	v-rel reticuloendotheliosis viral oncogene homologue B	2.009
212573_at	ENDOD1	Endonuclease domain containing 1	2.002

Ingenuity pathways analysis (IPA) showed that PR knock-down had the greatest effect on genes involved in cellular development, growth and proliferation. PR-induced genes were significantly enriched in two functional networks: (i) cellular development, growth proliferation and death (score 17 focus molecules 12); and (ii) cellular development, growth and proliferation, DNA replication, recombination and repair (score 15 focus molecules 11). Conversely, genes repressed by PR were significantly involved in three networks: (i) cell to cell signalling, inflammatory response, cellular movement (Score 21 focus molecules 15); (ii) cancer, cellular development, haematological system development (score 17, focus molecules 13); and (iii) skeletal muscular system development and function (score 15 focus molecules 12).

To explore the interactions between inflammatory signals and PR, myocytes in culture were incubated for 96 hrs with PR specific siRNA or non-targeting control siRNA, and then treated with MPA for a further 24 hrs. In the last 6 hrs of culture, cells were incubated with IL-1β (1 ng/ml) or vehicle control. We then compared the gene expression profiles of cells incubated with and without IL-1β in the absence of PR knock-down, i.e. transfected with non-targeting siRNA and in the presence of PR knock-down.

### IL-1β activates myometrial inflammation

In cells exposed to non-targetting siRNA, IL-1β significantly regulated 5160 probe sets (*P* < 0.05 with correction for multiple testing). Upon elimination of unannotated sequences and duplicate probe sets, 1440 (57%) and 1892 (43%) unique gene sequences were found to be up- and downregulated respectively. The expression of two genes, *CXCL2* and *CXCL3*, increased in excess of a 1000-fold. An additional 14 genes, mostly inflammatory in action, increased in expression ≥100-fold and 40 additional genes between 99- and 20-fold (Table 4). Of the 1892 repressed genes, 465 were inhibited ≥2-fold, which included 127 and 46 transcripts regulated ≥3 and 5-fold respectively (Table 5). The two probes complementary to PR mRNA on the array showed a 1.6- and 4.8-fold reduction in total PR transcript levels in response to IL-1β stimulation (*P* < 0.0002). As anticipated, IPA confirmed that IL-1β signalling has the greatest effect on gene networks involved in inflammation, immunity, anti-microbial response and NF-κB signalling.

**Table 4:** Unique genes whose expression was increased 20-fold or more by interleukin 1-beta. (In each case *n* = 4, *P* < 0.0005, Adjusted *P* < 0.05)

ID	Gene symbol	Gene title	Fold change in expression
209774_x_at	CXCL2	Chemokine (C-X-C motif) ligand 2	1504.177
207850_at	CXCL3	Chemokine (C-X-C motif) ligand 3	1008.501
205476_at	CCL20	Chemokine (C-C motif) ligand 20	491.925
211506_s_at	IL8	Interleukin 8	474.416
204470_at	CXCL1	Chemokine (C-X-C motif) ligand 1	465.483
204533_at	CXCL10	Chemokine (C-X-C motif) ligand 10	417.043
202638_s_at	ICAM1	Intercellular adhesion molecule 1	362.152
223484_at	C15orf48	Chromosome 15 open reading frame 48	311.712
202510_s_at	TNFAIP2	Tumour necrosis factor, alpha-induced protein 2	278.746
205207_at	IL6	Interleukin 6 (interferon, beta 2)	205.52
214974_x_at	CXCL5	Chemokine (C-X-C motif) ligand 5	197.62
202643_s_at	TNFAIP3	Tumour necrosis factor, alpha-induced protein 3	172.438
208075_s_at	CCL7	Chemokine (C-C motif) ligand 7	170.853
203868_s_at	VCAM1	Vascular cell adhesion molecule 1	144.993
205067_at	IL1B	Interleukin 1, beta	144.646
204748_at	PTGS2	Prostaglandin-endoperoxide synthase 2	103.175
206336_at	CXCL6	Chemokine (C-X-C motif) ligand 6	95.875
216598_s_at	CCL2	Chemokine (C-C motif) ligand 2	93.106
225516_at	SLC7A2	Solute carrier family 7, member 2	82.812
207442_at	CSF3	Colony stimulating factor 3 (granulocyte)	77.819
210538_s_at	BIRC3	Baculoviral IAP repeat-containing 3	73.068
205681_at	BCL2A1	BCL2-related protein A1	72.539
204798_at	MYB	v-myb myeloblastosis viral oncogene homologue (avian)	64.656
204614_at	SERPINB2	Serpin peptidase inhibitor, clade B, member 2	53.144
209706_at	NKX3-1	NK3 homeobox 1	51.161
205680_at	MMP10	Matrix metallopeptidase 10 (stromelysin 2)	48.891
214038_at	CCL8	Chemokine (C-C motif) ligand 8	48.42
217590_s_at	TRPA1	Transient receptor potential cation channel, subfamily A, member 1	47.185
215223_s_at	SOD2	Superoxide dismutase 2, mitochondrial	46.044
222549_at	CLDN1	Claudin 1	44.584
205266_at	LIF	Leukaemia inhibitory factor	41.476
220091_at	SLC2A6	Solute carrier family 2, member 6	40.655
205027_s_at	MAP3K8	Mitogen-activated protein kkk 8	36.936
205013_s_at	ADORA2A	Adenosine A2a receptor	36.636
213524_s_at	G0S2	G0/G1switch 2	36.454
229437_at	BIC	BIC transcript	36.36
209493_at	PDZD2	PDZ domain containing 2	35.692
202357_s_at	C2///CFB	Complement component 2 factor B	35.285
823_at	CX3CL1	Chemokine (C-X3-C motif) ligand 1	35.275
204224_s_at	GCH1	GTP cyclohydrolase 1	34.613
204273_at	EDNRB	Endothelin receptor type B	34.355
207386_at	CYP7B1	Cytochrome P450, 7B1	34.197
210133_at	CCL11	Chemokine (C-C motif) ligand 11	32.614
231779_at	IRAK2	Interleukin-1 receptor-associated kinase 2	31.006
235122_at	HIVEP3	HIV type I enhancer binding protein 3	28.811
1555759_a_at	CCL5	Chemokine (C-C motif) ligand 5	28.427
228186_s_at	RSPO3	R-spondin 3 homologue (Xenopus laevis)	27.918
218810_at	ZC3H12A	Zinc finger CCCH-type containing 12A	26.17
203180_at	ALDH1A3	Aldehyde dehydrogenase 1 A3	25.876
207510_at	BDKRB1	Bradykinin receptor B1	23.079
225316_at	MFSD2	Major facilitator superfamily domain 2	22.964
206924_at	IL11	Interleukin 11	22.963
205798_at	IL7R	Interleukin 7 receptor	22.495
205619_s_at	MEOX1	Mesenchyme homeobox 1	21.495
202902_s_at	CTSS	Cathepsin S	20.715
206432_at	HAS2	Hyaluronan synthase 2	20.527

**Table 5:** Unique genes whose expression was decreased 5-fold or more by interleukin 1-beta. (In each case *n* = 4, *P* < 0.0005, Adjusted *P* < 0.05)

ID	Gene symbol	Gene title	Fold change in expression
201009_s_at	TXNIP	Thioredoxin interacting protein	−26.955
226069_at	PRICKLE1	Prickle homologue 1 (Drosophila)	−11.959
238029_s_at	SLC16A14	Solute carrier family 16, member 14	−11.46
209582_s_at	CD200	CD200 molecule	−10.727
204424_s_at	LMO3	LIM domain only 3 (rhombotin-like 2)	−10.248
225171_at	ARHGAP18	Rho GTPase activating protein 18	−10.094
221911_at	ETV1	Ets variant 1	−9.948
220002_at	KIF26B	Kinesin family member 26B	−9.665
239710_at	FIGN	Fidgetin	−9.57
225548_at	SHROOM3	Shroom family member 3	−9.142
235085_at	PRAGMIN	Homologue of rat pragma of Rnd2	−8.716
229674_at	SERTAD4	SERTA domain containing 4	−8.512
206528_at	TRPC6	Transient receptor potential cation channel, C6	−8.502
227812_at	TNFRSF19	Tumour necrosis factor receptor superfamily, 19	−8.113
226492_at	SEMA6D	Sema domain, transmembrane domain (TM), and cytoplasmic domain, 6D	−7.632
206448_at	ZNF365	Zinc finger protein 365	−7.578
233533_at	KRTAP1-5	Keratin associated protein 1-5	−7.45
235591_at	SSTR1	Somatostatin receptor 1	−7.312
1552508_at	KCNE4	Potassium voltage-gated channel, Isk- 4	−7.278
219935_at	ADAMTS5	ADAM metallopeptidase with thrombospondin type 1 motif, 5	−7.264
242396_at	LOC100132798	Full length insert cDNA clone ZD42A11	−6.852
225977_at	PCDH18	Protocadherin 18	−6.798
229092_at	NR2F2	Nuclear receptor subfamily 2, group F, member 2	−6.79
222853_at	FLRT3	Fibronectin leucine rich transmembrane protein 3	−6.775
229114_at	GAB1	GRB2-associated binding protein 1	−6.536
235956_at	KIAA1377	KIAA1377	−6.523
235476_at	TRIM59	Tripartite motif-containing 59	−6.494
209292_at	ID4	Id-related helix-loop-helix protein Id4	−6.343
241752_at	SLC8A1	Solute carrier family 8, member 1	−6.338
218087_s_at	SORBS1	Sorbin and SH3 domain containing 1	−6.157
207233_s_at	MITF	Microphthalmia-associated transcription factor	−6.111
242794_at	MAML3	Mastermind-like 3 (Drosophila)	−6.049
225816_at	PHF17	PHD finger protein 17	−5.908
243140_at	ACTA2	Actin, alpha 2, smooth muscle, aorta, transcript variant 1	−5.816
231881_at	CALD1	Caldesmon 1	−5.794
219619_at	DIRAS2	DIRAS family, GTP-binding RAS-like 2	−5.739
228821_at	ST6GAL2	ST6 beta-galactosamide alpha-2,6-sialyltranferase 2	−5.678
226677_at	ZNF521	Zinc finger protein 521	−5.61
222662_at	PPP1R3B	Protein phosphatase 1, regulatory (inhibitor) subunit 3B	−5.588
209815_at	PTCH1	Patched homologue 1 (Drosophila)	−5.508
204338_s_at	RGS4	Regulator of G-protein signalling 4	−5.377
203373_at	SOCS2	Suppressor of cytokine signalling 2	−5.312
205304_s_at	KCNJ8	Potassium inwardlyrectifying channel, J8	−5.28
209829_at	FAM65B	Family with sequence similarity 65, member B	−5.164
204529_s_at	TOX	Thymocyte selection-associated high mobility group box	−5.051
223599_at	TRIM6	Tripartite motif-containing 6	−5.042

### IL-1β inhibits PR expression and microarray validation

The array analysis indicated that IL-1β inhibits *PR* expression in human myocytes. To validate the array, we performed Western analysis to examine the regulation of PR upon MPA and IL-1β stimulation in four independent primary myocyte cultures. IL-1β caused a significant reduction in the expression of both PR-B and PR-A upon 6 and 24 hrs of stimulation (Fig. 1C). This was associated with increased phosphorylation of p65, reflecting NF-κB activation, which peaked at 15 min., but persisted after 6 and 24 hrs (Fig. 1B). We further validated a set of selected genes regulated in a PR- and/or IL-1β-dependent manner by RTQ-PCR. These were selected arbitrarily in three groups: genes upregulated by both PR-knock-down and IL-1β stimulation; *MMP1*, *MMP3*, *MMP10:* genes upregulated by PR-knock-down; *COLEC12*, *STMN2*, *SCRA3*, *RXFP1*; and genes downregulated by PR-knock-down *THBS1*, *KCNJ2*, *COL12A1* and *TRPC6* (Fig. 1D). For these genes, the pattern of expression measured by RTQ-PCR analysis was in keeping with the array data.

### Activation of myometrial inflammation antagonizes PR-induced gene expression

**Fig 2:**
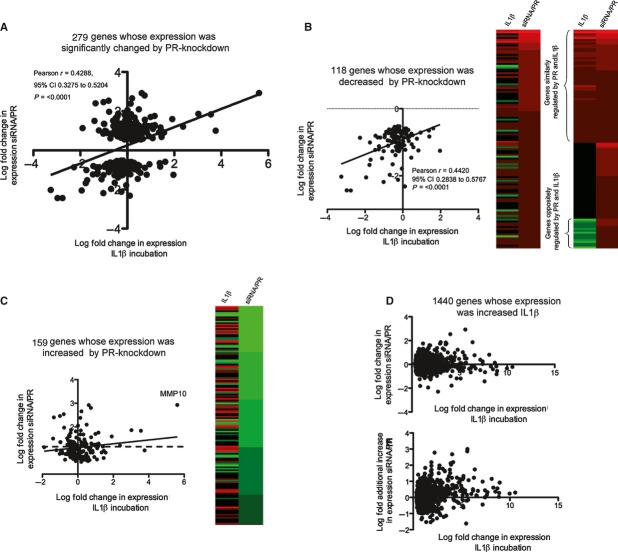
(A) Correlation between the effect of incubation with IL1b and siRNA PR-knock-down upon all 276 genes whose expression was found to be regulated by PR (*r* = 0.4288, *P* < 0.0001). (B) Correlation between the effect of incubation with IL1b and siRNA PR-knock-down upon the 118 genes whose expression was found to be decreased by PR-knock-down (*r* = 0.44, *P* < 0.0001). Heatmaps compare effect of IL1b and siRNA PR-knock-down organized by (left panel) descending effect of siRNA PR-knock-down and (right panel) into gene groups similarly and oppositely regulated by IL1b and siRNA PR-knock-down. (C) Correlation between the effect of incubation with IL1b and siRNA PR-knock-down upon the 158 genes whose expression was found to be increased by PR-knock-down. Dotted line shows correlation if MMP10 is omitted from analysis. Heatmap compares effect of IL1b and siRNA PR-knock-down organized by descending effect of siRNA PR-knock-down. (D) Correlation between the effect of incubation with IL1b and siRNA PR-knock-down upon the 1440 genes whose expression was found to be increased by IL1b. (E) Correlation between the effect of incubation with IL1b and siRNA PR-knock-down in the presence of IL1b upon the 1440 genes whose expression was found to be increased by IL1b.

We next tested if inflammatory signal intermediates, activated by IL-1β, repress PR-dependent gene expression in human myocytes. If activation of myometrial inflammation causes ‘functional progesterone withdrawal’, then it would be expected that IL-1β stimulation would have similar effects on PR-dependent gene expression as knock-down of the receptor. We therefore cross-referenced the effects of IL-1β on the expression of the 279 unique genes regulated upon PR-knock-down. Although we found a significant positive correlation between the overall effect of PR silencing on myometrial gene expression and IL-1β stimulation this correlation was weak (Fig. 2A, Pearson's *r* = 0.4288, 95% confidence interval: 0.33–0.52, *P* < 0.0001).

We then focused on those genes whose expression was repressed by PR knock-down, thus induced in a PR-dependent fashion. This showed a correlation with IL-1β-dependent gene expression (Fig. 2B, Pearson's *r* = 0.4420, 95% confidence interval: 0.28–0.58; *P* < 0.0001). The correlation became stronger if the analysis was confined to those genes repressed ≥2-fold upon PR knock-down (Pearson's *r* = 0.52, 95% confidence interval 0.27–0.7, *P* < 0.003; data not shown).

Heatmapping (Fig. 2B) demonstrated a clear cassette of genes whose expression was repressed by PR knock-down (thus induced in a PR-dependent fashion), and also repressed by IL-1β which are therefore co-regulated. If adjustment is made for multiple comparisons then 42 of 118 genes (22%) repressed by PR knock-down were also significantly repressed by IL-1β (at *P* < 0.05). However, a further 19 genes were also repressed by IL-1β at *P* < 0.05 where single testing only was taken into account making a total of 61 of 188 genes (32%).

When those genes were analysed whose expression increased upon PR knockdown (i.e. inhibited by PR), the correlation with the IL-1β-dependent transcriptional response, although still significant was very weak (Fig. 2C, Pearson's *r* = 0.35, 95% confidence interval: 0.2–0.48, *P* < 0.0001) and was entirely dependent upon the strong induction of a single gene, *MMP10*, in response to IL-1β and upon PR-knock-down. Omitting MMP10 from the analysis indicated that IL-1β-dependent activation of myometrial inflammation has no significant effect on the ability of PR to repress specific gene sets (*P* = 0.57).

### PR does not repress pro-inflammatory gene networks induced by IL-1β

We next hypothesized that PR, liganded or not, may serve to repress inflammatory genes in otherwise unstimulated myocytes. If so, PR silencing in the absence of inflammatory stimulation should be sufficient to regulate genes which are inducible by IL-1β. Alternatively, it is possible that PR only confers transcriptional repression of genes involved in myometrial inflammation upon exposure to inflammatory cues. If this hypothesis holds true then PR knock-down should further increase the expression of genes induced by IL-1β treatment.

To explore the first hypothesis, we correlated the effect of PR knock-down in unstimulated myocytes on expression of genes which would be activated upon IL-1β treatment. No correlation was found (Fig. 2D). Of 1440 genes induced by IL-1β in human myocytes, PR silencing significantly increased the expression of only 36 (0.025%) genes, and just 15 of these by ≥2-fold. We then explored the second hypothesis, but again no correlation was found (Fig. 2E). PR-knock-down failed to significantly augment the vast majority of genes induced by IL-1β stimulation. The expression of only 39 (0.027%) of 1440 IL-1β-induced genes was significantly enhanced upon prior PR silencing, with transcript levels of merely 22 of these upregulated by ≥2-fold.

In the absence of a ‘global’ anti-inflammatory role for PR, we speculated that this nuclear receptor may still be important for the repression of a discrete cassette of genes encoding key inflammatory mediators. We therefore focussed on those IL-1β upregulated genes whose expression increases upon PR silencing in both stimulated and unstimulated cells. Only seven such genes were identified from the microarray analysis (Table 6). Furthermore, for all but one gene, *MMP10*, the increase caused by IL-1β alone and additional increase in transcript levels caused by PR knock-down in the presence or absence of IL-1β was modest, albeit significant. One gene, MMP1, showed a similar pattern of expression to MMP10, but on the microarray analysis whilst the effect of IL-1β, and PR knock-down in the presence of IL-1β was significant, the effect of PR knock-down alone was significant for single testing but not when multiple testing was taken into account. We selected this gene for independent validation studies and found that, in those studies using qRT-PCR, the effects of IL-1β, PR knock-down alone and PR knock-down in the presence of IL-1β were all significant (Fig. 1D).

**Table 6:** Unique IL1b upregulated genes whose expression was increased in unstimulated cells by PR-knock-down (siRNA/PR FC i.e fold change), and further increased by PR-knock-down in the presence of IL1b (siRNA/PR + IL1b FC i.e fold change). (In each case *n* = 4, *P* < 0.0005, Adjusted *P* < 0.05)

ID	Gene Symbol	Gene name	Fold increase IL1β	Fold increase siPR *versus* control	Fold increase siPR + IL1β v IL1β alone
205680_at	MMP10	Matrix metallopeptidase 10	48.9	7.6	8
204475_at	MMP1	Matrix metallopeptidase 1	16	5.8*	6.4
218330_s_at	NAV2	Neuron navigator 2	2.2	3.4	2.5
209960_at	HGF	Hepatocyte growth factor	6.2	2.6	2.8
213562_s_at	SQLE	Squalene epoxidase	2.7	2.2	1.8
223395_at	ABI3BP	ABI family, 3 (NESH) binding protein	1.6	2.2	2.3
213725_x_at	XYLT1	Xylosyltransferase I	1.6	2.1	1.9

* Significant for single but not multiple testing.

## Discussion

Understanding the mechanism of the onset of human parturition is critical for the design of strategies to predict and prevent preterm birth. However, this requires more than simply understanding the mechanisms of contractions. Although a range of drugs is available which are intended to inhibit uterine contractions their clinical effectiveness is disappointing. The ‘tocolytic’ reactive management of preterm labour is giving way to a strategy based upon prediction and prevention, however, the only agent which currently shows promise in preventing the onset of preterm labour is progesterone. Progesterone appears to be effective in some woman at high risk of preterm birth with a singleton pregnancy, but does not reduce the risk of prematurity in multiple pregnancy [12]. Further development of progestational agents and similar drugs for this indication requires a better understanding of the function of progesterone and its receptor within the uterus.

We have modelled PR-mediated functional progesterone withdrawal by using siRNA knock-down of PR in the presence of a synthetic progesterone. Comparisons were made between siRNA/PR and control always in the presence of synthetic progesterone. We did not compare gene expression between cells in the presence or absence of progesterone as there is no withdrawal of progesterone in association with human labour and therefore, *in vivo*, there is always PR ligand present. We have used MPA rather than natural progesterone because of its greater long-term stability in culture. MPA has been reported to have glucocorticoid receptor activity [13], but we have found that this also applies to natural progesterone [14]. Use of siRNA requires the use of passaged cells in culture, as it is not possible to obtain sufficient cells numbers for experimental purposes without passage. Such models are widely used in investigation of myometrial cell function, however, they do risk the possibility that cultures become enriched for rapidly dividing fibroblast cells and there is loss of the extracellular matrix integrity and cell-matrix interaction of the intact tissue. We have confirmed that our cell cultures after passage and MPA incubation are alpha-actin and oxytocin receptor positive and that expression of PR and the PR-A:PR-B ratio is similar to that seen in fresh myometrial tissue. A caveat to this, however, is that we have found some patient to patient variability in PR-A:PR-B ratio in both myometrial tissue taken at pre-labour caesarean section and subsequent cell culture which may be dependent upon how close the individual woman is to the onset of labour.

Progesterone is widely thought to act principally to repress contractions through repressing ‘contraction-associated proteins’. However, it is now clear that progesterone plays a more complex role in myometrial physiology during pregnancy. In the rat, progesterone is involved in phenotypic modulation of myocytes during the synthetic phase of myometrial differentiation in the last third of pregnancy during which there is myometrial hypertrophy and synthesis and deposition of interstitial matrix [15]. We found that in human myocytes PR regulates a relatively small number of genes. We did not see significant down-regulation of classic ‘contraction-associated proteins’ by PR. The major effect of PR upon gene expression was upon genes concerned with cellular development, growth and proliferation. PR also highly upregulates two ion channels; transient receptor potential canonical-6 (TRPC-6) and the inward-rectifier potassium ion channel KCNJ2. TRPC channels in general mediate store-operated calcium entry. Expression of TrpC1, TrpC3, TrpC4 and TrpC6 has been demonstrated in human myometrium [16]. Tonic stretch co-regulates calcium entry pathways and TRPC3 and TRPC4 expression suggesting that these receptors play a role in increasing contractility [17]. Trp6, however, differs in that it mediates non-store operated calcium entry and TrpC6 has been shown to play an essential role in cellular proliferation in a range of cell types [18, 19].

Fibronectin leucine rich transmembrane protein-3 (FLRT-3), and thrombospondin 1 (THBS1) were found to be highly upregulated by PR whilst matrix metallopeptidase 10 (MMP-10) was highly repressed. FLRT3 is a member of the fibronectin leucine rich transmembrane protein family which play important roles in cell matrix adhesion. THBS1 is an adhesive glycoprotein which binds fibrinogen, fibronectin, laminin, collagen and integrins and also mediates cell-to-matrix interactions. MMP-10 is a member of the matrix metalloproteinase family which is involved in the breakdown of extracellular matrix. Collectively, these data point to a role for PR regulated genes in the synthesis and maintenance of interstitial matrix in the human. PR therefore probably acts during pregnancy more to regulate the growth and development of the uterus than to directly inhibit contractions, which is consistent with progesterone having efficacy as a prophylactic agent, but not as a tocolytic agent.

Our principal purpose was to shed light on the inter-relationship between PR and the myometrial inflammation. Parturition can be thought of, at least in part, as inflammatory in nature as it is associated with upregulation of prostaglandin, cytokine and chemokine synthesis with the uterus and with an influx of macrophages and lymphocytes into the myometrium and cervix [20]. Although the inflammatory cell infiltrate may be a source of cytokines within the labouring uterus it is clear that myometrium itself is a major source of inflammatory mediators [21]. IL-1β concentrations rise within the uterus in association with both term and preterm labour [22]. We activated myometrial inflammation by incubation with IL-1β which activates both NF-κB and AP-1 leading to upregulation of a wide variety of inflammatory genes which have been shown to be associated with parturition [23, 24]. As would be expected we found that IL-1β increased expression of genes involved in immunity, inflammation, anti-microbial response and NF-κB signalling.

Seminal studies of the endocrinology of parturition in sheep and rodent models suggested that progesterone withdrawal is an early initiating factor leading to subsequent upregulation of ‘inflammatory’ type mediators, most importantly prostaglandins. This would suggest that progesterone acts to inhibit the inflammatory biochemistry of parturition. One hypothesis of the mechanism of human parturition suggests that the activation of inflammation occurs late in the process of parturition and that its principal role is the involution of the uterus following delivery. However, there is growing evidence that inflammation is an early initiating event in human parturition which begins before the onset of contractions. Activation of NF-κB within the uterus appears to play an important role in the onset of labour, and in the myometrium principally regulates a group of immune/inflammation associated genes [8, 21, 25].

Condon *et al*. [5] have shown no activation of NF-κB in myometrium in the third trimester of pregnancy remote from the onset of labour. However, in a study in which we compared tissue samples from women taken either before or after labour at term we found that that NF-κB is active in myocytes in both the upper and lower segment of the uterus prior to the onset of labour at term [21]. This, taken together with data which shows that inhibition of NF-κB in the mouse prevents normal term labour [8] suggest that NF-κB begins to be activated in myometrium close to, but prior to, the onset of labour at term. This concept is supported by the results of mathematical modelling studies [26], which explored three main hypotheses for the activation of the human uterus at labour: functional progesterone withdrawal; inflammatory stimulation; and oxytocin receptor activation. These were modelled using gene expression data in pre-labour myometrial samples using directed graphs. It was found that inflammatory activation as a primary event in parturition was highly likely, progesterone withdrawal, as a primary event, was less likely but plausible, and that oxytocin receptor mediated initiation was unlikely.

We considered three non-mutually exclusive hypotheses concerning the interaction between inflammation and PR in myocytes. These were that inflammation acts to repress the function of PR; that liganded PR acts to repress basal expression of inflammatory mediators; or that liganded PR acts to repress stimulated expression of inflammatory mediators in myocytes. Our data shows that activation of inflammation does act to inhibit PR function, but, in general only in relation to genes which are upregulated, not downregulated by PR. Specifically, we identified a cassette of genes, representing some 20–30% of genes upregulated *via* PR that are downregulated by activation of inflammation. This is possibly a consequence of the different ways in which PR interacts with co-factors and the promoters of genes which are either up- or downregulated. There was, however, no general anti-inflammatory effect of activated PR either upon basal or stimulated expression of inflammatory mediators. We identified a few genes which were oppositely regulated by inflammation or PR, most significantly the matrix-metalloproteinases, MMP10 and MMP3, but not cytokines, chemokines or ‘contraction-associated proteins’. The genes neuron navigator 2, hepatocyte growth factor, squalene epoxidase, ABI family, 3 (NESH) binding protein and xylosyltransferase I were each found to be oppositely regulated by inflammation or PR, but the overall effect of either IL-1β or PR-knock-down was very small when compared with those effects in relation to MMP1 or MMP10. It is doubtful that the opposite regulation of these six genes by inflammation or PR is of significance in the context of parturition. Interestingly, although progesterone has been shown to inhibit IL-1β stimulated expression of prostaglandin-endoperoxide synthase type-2 and interleukin-8 in human myocytes, uterine fibroblasts and amnion cells [11, 27] in the current study PR-knock-down did not enhance the IL-1β stimulated expression of either of these genes suggesting that the effect of progesterone in this context is not mediated *via* PR. Progesterone itself may nevertheless have a greater anti-inflammatory role within the uterus, but mediated through receptors other than PR [14].

Our data are consistent with activation of inflammation being a significant mediator of functional-progesterone withdrawal in the context of the PR mediated action of progesterone. There are several mechanisms, which may act together, by which inflammation may repress PR function. In the present study, we found that IL-1β caused a significant downregulation in expression of PR itself although not a change in the ratio of PR-B to PR-A. Mesiano *et al*. [1, 2] have suggested that it is a change in the ratio of the PR-B to PR-A isoforms of PR which leads to functional-progesterone withdrawal and have shown that this may be mediated by increased prostaglandin synthesis. As would be expected, we found that incubation with IL-1β leads to a large increase in prostaglandin-endoperoxide synthase type-2 expression. However, it is unlikely that inhibition of PR synthesis is the sole mechanism since at the 6-hr time point examined PR expression had reduced by only one-third. Furthermore, the effect of IL-1β upon PR regulated genes was only upon those upregulated, suggesting a mechanism other than simple receptor withdrawal. That it is likely that NF-κB is activated within the uterus early in the biochemical events of labour suggests that the negative interaction between NF-κB p65 and PR may also represent an important mechanism of functional-progesterone withdrawal. Activation of NF-κB leads to increased synthesis of its own p65 (RelA) subunit and to increased synthesis of IL-1β and so to a positive feed forward loop in which there is both synthesis and activation (phosphorylation) of p65 and therefore repression of PR. Our data supports inhibition of PR by inflammation in general, or NF-κB specifically, but suggests that the negative interaction between NF-κB p65 and PR does not function in myometrium in the opposite direction as we found no evidence of PR acting to repress NF-κB regulated genes.

Much of what is currently known about the endocrinology and biochemistry of parturition has come from animal models, however, human parturition is unusual in that circulating progesterone levels do not fall until after delivery [2]. Ethical considerations mostly limit *in vivo* experiments in humans to observations. Studies involving manipulation of endocrine or inflammatory factors in humans are very difficult to undertake *in vivo* and so need to be modelled *in vitro*. Inevitably, no model can take into account all of the complex endocrine, paracrine and mechanical factors which interact in the uterus *in vivo*. The model that we developed for this study was made as simple as possible to answer our key questions: what are the genes regulated by PR in the myocyte in the presence of progesterone and how does PR-function and inflammation interact. Overall the data that we present here support the concept that progesterone acts through PR in human myocytes to mediate cellular development, growth and proliferation and maintain the interstitial matrix, but that progesterone does not play a significant anti-inflammatory role *via* PR. Conversely inflammation does act to generally inhibit expression of genes which are upregulated by PR. This is consistent with a hypothesis for the mechanism of human parturition in which activation of inflammatory mediators, likely through activation of NF-κB, is an early event preceding ‘functional progesterone withdrawal’.
